# The ontogenetic dietary shift from non‐dangerous to dangerous prey in predator‐eating predators under capture risk

**DOI:** 10.1002/ece3.9609

**Published:** 2022-12-08

**Authors:** Yuya Suzuki, Mito Ikemoto, Tomoyuki Yokoi

**Affiliations:** ^1^ Laboratory of Conservation Ecology, Graduate School of Life and Environmental Sciences University of Tsukuba Ibaraki Japan; ^2^ The United Graduate School of Agricultural Sciences Kagoshima University Kagoshima Japan; ^3^ Biodiversity Division National Institute for Environmental Studies Tsukuba Japan

**Keywords:** dietary specialist, natural diet, ontogeny, prey‐capturing traits, prey–predator size relationship

## Abstract

Evaluating the patterns and generality of ontogenetic dietary shifts (ODSs) contributes to understanding prey–predator interactions and food web dynamics. Numerous studies have focused on predators that target distinctively lower trophic‐level organisms. However, the ODS of predators that routinely prey on organisms at similar trophic levels (i.e., predator‐eating predators) have been neglected in ODS research. The ODS patterns of predator eaters may not fit into conventional frameworks owing to constraints of potential capture risk (e.g., deadly counterattack from prey) and body size. We aimed to reveal the ODS patterns of predator eaters and determine whether the patterns were affected by body size and capture risk. Assuming that capture risk is a significant factor in ODS patterns, we expected: (1) juvenile araneophagic spiders to forage on non‐dangerous prey (insects) and capture larger non‐dangerous prey more frequently than dangerous prey (spiders); and (2) as they grow, their prey types will shift from non‐dangerous to dangerous prey because larger predators will be able to capture dangerous prey as the optimal food. As a result of field observations, we revealed that the major ODS pattern in these spiders changed from a mixed (both insect and spider) to a spider‐dominant diet. The model selection approach showed that this diet shift was partly due to predator size, and the relative importance of predator size was higher than the life stage per se and almost equal to species identity. In these spiders, the body size of spider prey tended to be smaller than that of insects when the predators were small, suggesting that capture risk may be a critical factor in determining the ODS patterns of these predators. Therefore, our study adds to the evidence that the capture risk is crucial in comprehensively understanding the mechanisms determining ODS patterns in natural systems.

## Graphical abstract

In this study, we aimed to reveal the ontogenetic dietary shifts (ODSs) patterns of predator eaters and determine whether the patterns are affected by body size and capture risk of dangerous prey (i.e., spiders), by targeting araneophagic spiders. Field surveys revealed that the major ODS pattern in these spiders changed from a mixed (both insects and spiders) to a spider‐dominant diet, and the body size of spider prey eaten by the predators tended to be smaller than that of insects.

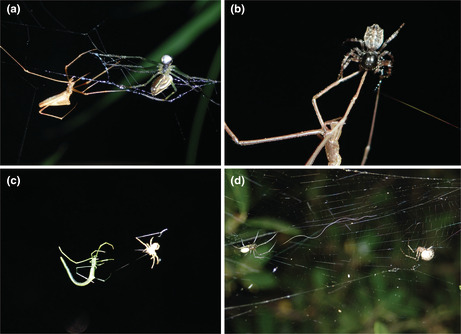

## 1 INTRODUCTION

Ontogenetic dietary shift (ODS), meaning changes in the diet along the life span of predators, is widespread in various prey–predator systems (Alejandro et al., 1998; Lowe et al., 1996; Mushinsky et al., 1982; Plummer & Goy, 1984). Many studies have described the patterns of ODS and attempted to reveal the underlying factors (Sánchez‐Hernández et al., 2019) because the combinations and intensity of prey–predator interactions characterize food web structure and dynamics (Allesina et al., 2008; Krause et al., 2003; Petchey et al., 2008). There is increasing evidence that the body sizes of prey and predators are cardinal factors affecting ODS. This is because predation capability is strongly limited by body size. Consequently, predators tend to select larger prey as their bodies grow larger (Brose, 2010; Brose et al., 2006; Cohen et al., 1993; Erickson & Morse, 1997; Sloggett, 2008). For example, the prey of predators with a wide diet breadth, such as fish and snakes, are strongly restricted by the relationships between the body size of the prey and the mouth size of the predator due to their gape or swallow‐feeding mechanisms (Costa, 2009; Jensen et al., 2012; Mori & Vincent, 2008; Shine & Sun, 2003; Tsai et al., 2016). Concerning predators with a narrow diet breadth, some species deal with this size constraint by switching among distinct prey taxa with different prey sizes (Yeargan & Quate, 1996). Other species overcome size constraints (capturing prey much larger than themselves) by using specialized predatory behavior (Nakazawa et al., 2013; Ohba & Tatsuta, 2015). These studies have suggested that ODS patterns vary among prey–predator systems.

Most ODS studies have examined prey–predator relationships in which predators forage on prey distinctively lower trophic levels, such as herbivores, omnivores, or detritivores (Sánchez‐Hernández et al., 2019). On the other hand, some predators routinely forage on organisms at a more similar trophic level in nature, such as ophiophagous (i.e., snake‐eating) snakes (Bhaisare et al., 2010) and araneophagic (i.e., spider eating) spiders (Jackson, 1985; Jackson et al., 2002; Pekár, Coddington, et al., 2011; Pekár, Sobotník, et al., 2011). Feeding on predators may be beneficial in terms of nutrient efficiency (Fagan et al., 2002; Polis, 1981; Raubenheimer et al., 2007). However, it increases the risk of deadly counterattacks from prey. For example, araneophagic spiders are sometimes lethally injured by the defensive behavior of spider prey (Harland & Jackson, 2006; Michálek, Lubin, & Pekár, 2019). Such a risk is hereafter referred to as “capture risk” for predators, and predators that feed on predators routinely are referred to as “predator‐eating predators.” Owing to potential capture risk and size constraints, the ODS patterns of predator‐eating predators may show different patterns from those of conventional ODS frameworks. However, the relationships between capture risk and prey–predator size relationships are poorly understood, with several studies only suggesting that predators that eat predators occasionally (i.e., eat herbivores mainly) seem to prefer safer and smaller prey than dangerous and larger ones (Elbroch et al., 2016; Hayward et al., 2006; Mukherjee & Heithaus, 2013). Therefore, examining ODS patterns and size dependencies in predator‐eating predators is crucial to understand the patterns and mechanisms of ODS comprehensively.

In this study, we provided the first empirical evidence that the ODS of predator‐eating predators may be distinct from ordinary predators that mainly feed on lower trophic levels. We targeted four araneophagic spiders: three from the family Theridiidae and one from the family Araneidae. In this study, “araneophagic” spiders include not only stenophagous specialists (i.e., natural diet is restricted to spiders) but also euryphagous specialists: predators routinely forage other spiders by specialized behavior, such as web invasion, and also accept insects as prey (e.g., Jackson & Blest, 1982; Jackson & Wilcox, 1994; Michálek, Lubin, & Pekár, 2019; Pekár & Toft, 2015). The study species sympatrically occur in the same habitat, although prey‐capturing traits differ among them (see Section 2: Material and Methods for details). We defined spiders as dangerous prey and insects as non‐dangerous prey. Insect prey may include various dangerous species, such as aggressive hunters (e.g., mantids and wasps) and armored insects (e.g., spined caterpillars). Nonetheless, it is suggested in our preliminary field observations that araneophagic spiders never encounter or prey on such dangerous insect taxa. Instead, they may eat small, non‐aggressive, weaponless insects, such as detritivorous Diptera, in the field. We regarded these insects as non‐dangerous prey in this study because they never injured their predators. Here, we hypothesized that if capture risk is a significant factor, (1) juvenile araneophagic spiders would tend to forage on less dangerous prey (insects), and thus, their prey types would shift from insects (i.e., non‐dangerous) to spiders (i.e., dangerous prey) as the spiders grew, and (2) the body size of edible spider prey would be smaller than that of insects, especially when the spiders were juveniles. To confirm these hypotheses, we revealed the actual ODS patterns of araneophagic spiders as predator‐eating predators and analyzed whether the prey–predator size relationships depend on prey types (spiders or insects).

## 2 MATERIALS AND METHODS

### 2.1 Study species and prey‐capturing traits

**FIGURE 1:**
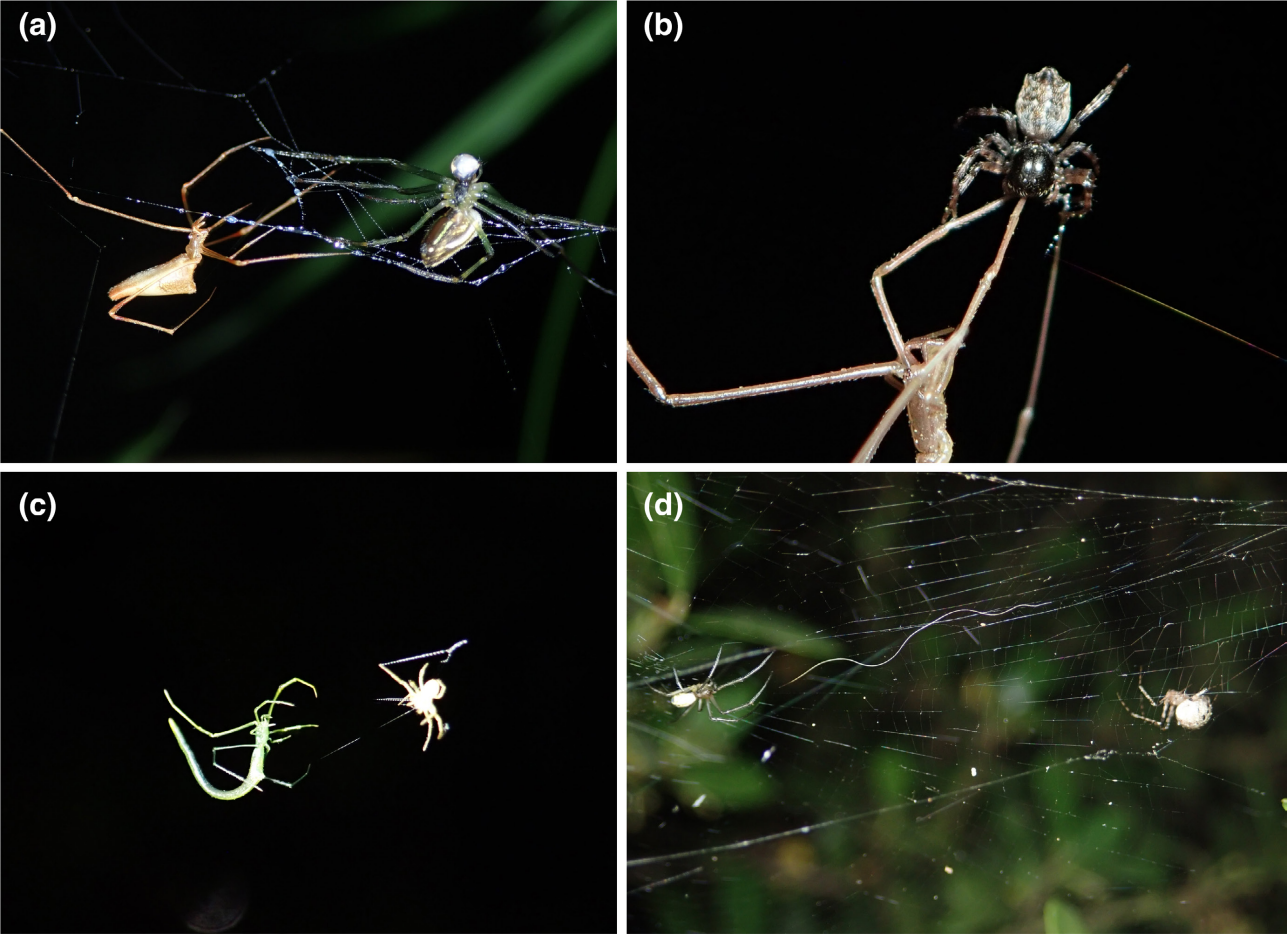
Study species and their prey‐capturing traits. (a) *Rhomphaea* sp. (left) throwing a sticky thread to *Leucauge celebesiana* (right). (b) *Chorizopes nipponicus* (upper) biting a leg of *Ariamnes cylindroaster* (lower). (c) *Ariamnes cylindrogaster* (left) capturing a crab spider (right) on its dry threads. (d) *Platnckina sterninotata* (right) walking on threads of the web of *Trichonephila clavata* (left).

The four study species, *Rhomphaea* spp., *Ariamnes cylindrogaster* Simon, 1889, *Platnickina sterninotata* (Bösenberg & Strand, 1906; Araneae: Theridiidae), and *Chorizopes nipponicus* Yaginuma, 1963 (Aranea: Araneidae), are widely distributed in mainland Japan and inhabit forests, woods, and bushes (Shinkai, 2006). They are arboreal and hang on branches, leaves, their threads, or other spider webs (Shinkai, 2006). Their prey‐capturing traits are characterized by prey‐capturing tools and prey‐searching locations. Prey‐capturing tools differed among the three theridiids and the araneids. Theridiids catch their prey by throwing silk with viscid droplets over the body of the prey (Figure 1a) and then wrapping it up with threads (Suzuki, 2020; Whitehouse, 1987). In contrast, *Chorizopes* bite the legs or body of their prey with fangs and inject venom (Eberhard, 1983; Figure 1b). Prey‐capturing locations differ between *Ariamnes* (Theridiidae) and the other spiders. *Ariamnes* spiders construct a simple web composed of a few non‐sticky threads and capture prey that accidentally walk on it (Figure 1c; Shinkai, 2006). In contrast, the other species invade webs and capture the hosts of the webs (Figure 1d).

### 2.2 Field observation

We conducted nocturnal line censuses in the forests of University of Tsukuba, Tsukuba city, Ibaraki, Japan, from April to October 2018, at intervals of 3–5 days (a total of 63 times). We placed two census lines with a length of 200 m at two sites: a secondary forest (36.117815 N, 140.099090 E; hereafter called “A”) and an arboretum (36.115551 N, 140.100620 E; hereafter called “B”), with a distance of 235 m between them. Vegetation at both sites is composed of sub‐tall trees (e.g., *Quercus* oak trees) and shrubs (e.g., *Aucuba japonica*, *Eurya japonica*, and *Cleyera japonica*). The forest floor of site A was covered with *Pleioblastus chino* bamboo grasses, while that of site B was regularly mowed.

Line censuses were conducted between 8:00 p.m. and 11:00 p.m. (3 h/day). When we found an araneophagic spider eating prey, we took photos of the spider and its prey using a digital camera (OLYMPUS STYLUS TG‐4; Japan) with a plastic ruler as a scale. The observed prey was immediately collected and preserved in 80% ethanol for identification. Araneophagic spiders were not collected to minimize the impact on their population size during the survey period. We also recorded the location where predation events occurred (on the web of the prey or line threads). In the laboratory, the sizes of the predator and prey (total length, in mm) were measured from the photographs using image processing software (ImageJ bundled with 64‐bit Java 1.8.0_112). Based on the absence or presence of genital organs, predator individuals were classified into two life stages: juveniles and adults. Non‐spider prey (less‐aggressive herbivorous and detritivorous insects) were observed and classified into order levels. Spider preys at the genus level were observed under a stereoscopic microscope (Nikon AZ100M).

### 2.3 Statistical analysis

All statistical analyses were performed using R ver. 4.0.2 and 4.2.0 software (R Development Core Team, 2020, 2022). The proportions of dangerous prey (spider prey) to all prey in the natural diet were compared among the four species or between juveniles and adults in each species using Fisher's exact probability test. Diet breadths in juveniles and adults of each araneophagic spider were evaluated using Shannon's diversity index (H′) (function diversity, package vegan; Oksanen et al., 2020). Spider prey species were classified at the family level, while other prey species were classified at the order level.

**FIGURE 2:**
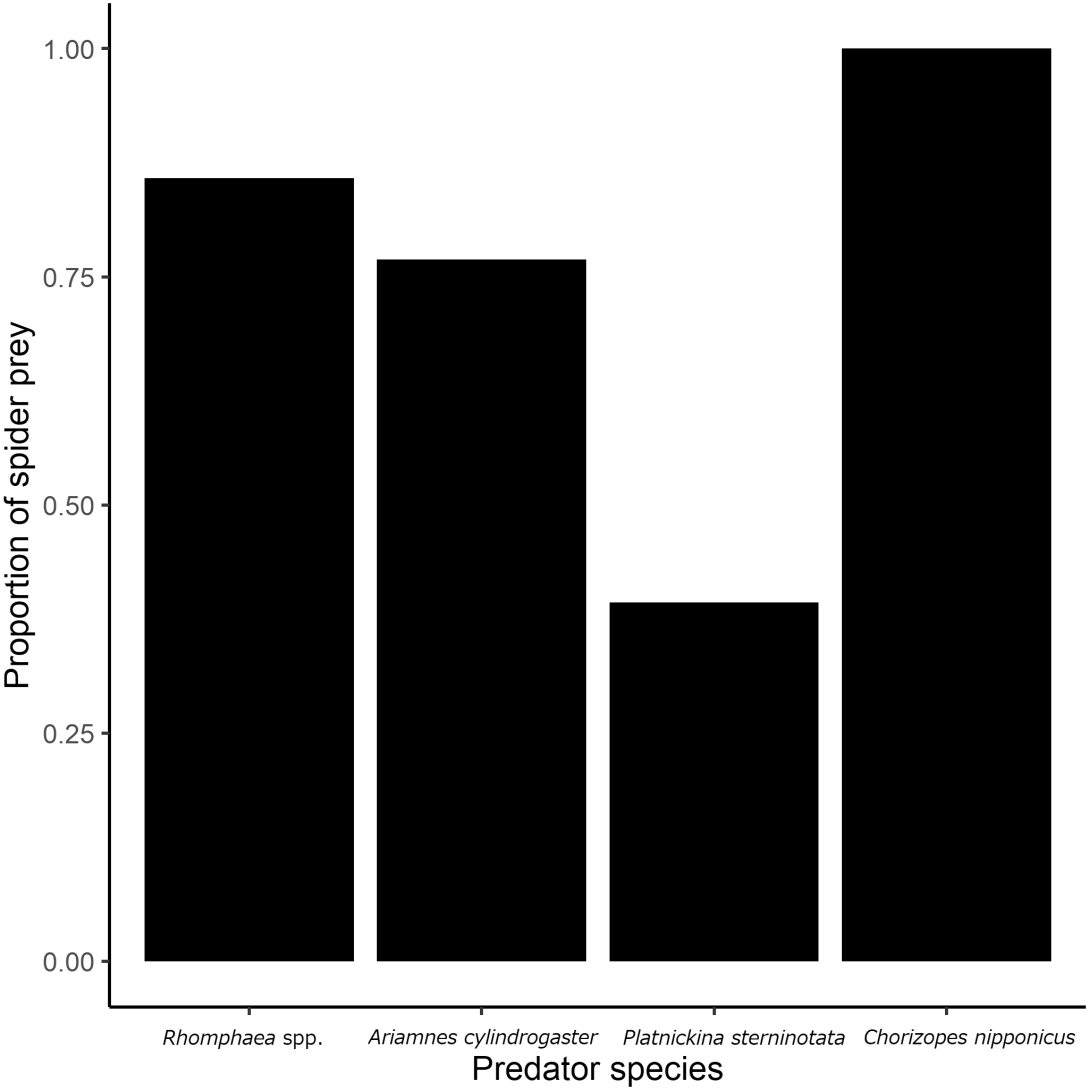
The proportion of spider prey in the diet of four araneophagic spiders.

We constructed GLMs about the proportion of predation on spider prey and prey–predator size relationships to assess whether capture risks can be a cardinal factor of ontogenetic diet shift in araneophagic spiders. In analyzing the proportion of spider prey, our main interest is whether predator size, which inherently relates to the capture risk, can influence the proportion of predation on spiders, which in turn can influence ontogenetic diet shift. Yet, other uncontrolled factors, including species identity and unknown effects concerning the life stage itself (e.g., stage‐dependent resource requirement), will interactively influence the results. Therefore, we built models using all possible combinations of the explanatory variables and selected the best models based on AICc (function dredge, package MuMIn; Barton, 2022). We regarded models with ΔAICc < 2.0 are the plausible models among all candidate models and applied model averaging of those top models to obtain parameter estimates for visualization (function model.avg, package MuMIn; Table 1). Additionally, we calculated the sum of the overall model weights of each explanatory variable (function sw, package MuMIn) to obtain an interpretation of their relative importance (Burnham & Anderson, 2002). Based on the above statistical procedure, we set predator size (total length), life stage (adults or juveniles), species of predator spiders, and those interactions as explanatory variables for the analysis of the proportion of spider prey (binary data: spider = 1, insect = 0), postulating binomial error distribution (logit link). The analysis of the relationships of body sizes between each araneophagic spider and prey was also conducted using the same statistical procedure. For this, the response variables were set as the observed prey size of each spider, and the explanatory variables were set as predator species, predator size, prey type (spider or not), and those interactions [gamma error distribution (log link)]. In the GLM analyses, we excluded *C.* *nipponicus* because this species feeds only on spiders throughout their life stages (Figure 2), and thus is inadequate to be treated as same as the other three species. Hence, the effect of predator size was examined only for the prey size of *C. nipponicus* [gamma distribution (log link)].

**TABLE 1:** The results of model selection for (a) the proportion of spider prey of the spiders that feed on both spiders and insects (GLM, binomial distribution) and (b) the prey size of the spiders that feed on both spiders and insects (GLM, gamma distribution).

(a)										
Model Rank	Predator size	Stage	Species	Predator size × Stage	Predator size × Species	Stage × Species	Predator size × Stage × Species	AICc	ΔAICc	AICc weight
1	+	+	+	+		+		461.47	0.00	0.27
2	+	+	+					461.82	0.35	0.23
3	+		+		+			462.04	0.57	0.20
4	+	+	+		+			462.20	0.73	0.19
5	+	+	+			+		463.12	1.64	0.12
Sum of weights	0.98	0.82	1.0	0.37	0.42	0.36	<0.01			
N	14	14	14	6	6	6	1			

(b)										
Model Rank	Predator size	Prey type	Species	Predator size × Prey type	Predator size × Species	Prey types × Species	Predator size × Prey type × Species	AICc	ΔAICc	AICc weight
1	+	+	+	+	+	+		2603.51	0.00	0.48
2	+	+	+		+	+		2604.18	0.68	0.34
3	+	+	+	+	+	+	+	2605.39	1.89	0.19
Sum of weights	1.0	1.0	1.0	0.66	1.0	1.0	0.18			
N	14	14	14	6	6	6	1			

*Note*: The candidate explanatory variables (gray color), attributes (white color) of the top models (ΔAICc < 2.0), and attributes of each explanatory variable (dark gray color) are shown. The models were ranked by AICc. + symbols represent that the corresponding variable was selected in the corresponding model. The sum of weights represents the relative importance of each explanatory variable (Burnham & Anderson, 2002). N shows the number of models in which the corresponding variables are involved because the sum of the weights of the explanatory variables is comparable only when the number of involved models is the same (Burnham & Anderson, 2002).

## 3 RESULTS

A total of 511 predation events were observed among the four study species. In most cases, araneophagic spiders preyed on other spiders or insects (*n* = 483/511: 94.5%), and intra‐ and interspecific predation between araneophagic spiders rarely occurred (intra‐specific – *n* = 9/511: 1.76%; interspecific – *n* = 19/511: 3.72%). Throughout the survey, juvenile predation events were observed more often than adults in *Rhomphaea* spp. (proportion of juveniles in the total number of observations: 60.7%) and *A. cylindrogaster* (72.0%). At the same time, juveniles were found less often in *P. sterninotata* (44.8%) and *C. nipponicus* (25.0%). The body size of adults tended to be larger than that of juveniles (Figure S1).

The proportion of spider preys significantly differed among the four species (Fisher's exact probability test, *p* < .05; Figure 2). *Chorizopes nipponicus* exclusively preyed on spiders, whereas *Rhomphaea* spp. (86%), *A. cylindrogaster* (77%), and *P. sterninotata* (39%) preyed on both spiders and non‐spiders (Figure 2). The proportions of spider prey were significantly higher in adults than juveniles of the latter three theridiids (Fisher's exact probability test, *p* < .05; Figure S2). Spider prey taxa of the four study species included various spider families from web‐building and cursorial species (Table S1, Figure S4). The diet breadth of adults was narrower than that of juveniles, except that of the spider prey family in *C. nipponicus* (Table S2).

The body size of most non‐spider prey was small, and no weapon‐like traits were observed (i.e., our assumption that non‐spider prey should be non‐dangerous was adequate). Specifically, Diptera was dominant in the non‐spider prey taxa (Table S1, Figure S3). Among dipteran prey, Nematocera flies, such as gall midges (Diptera: Cecidomyiidae) and fungus gnats (Diptera: Mycetophilidae), were most frequently eaten (accounting for 57% of non‐spider prey; Table S1, Figures S3 and S5). Non‐spider prey taxa also included Coleoptera (larva), Hymenoptera, Lepidoptera (larva), Homoptera, Psocoptera, and so on (Table S1).

**FIGURE 3:**
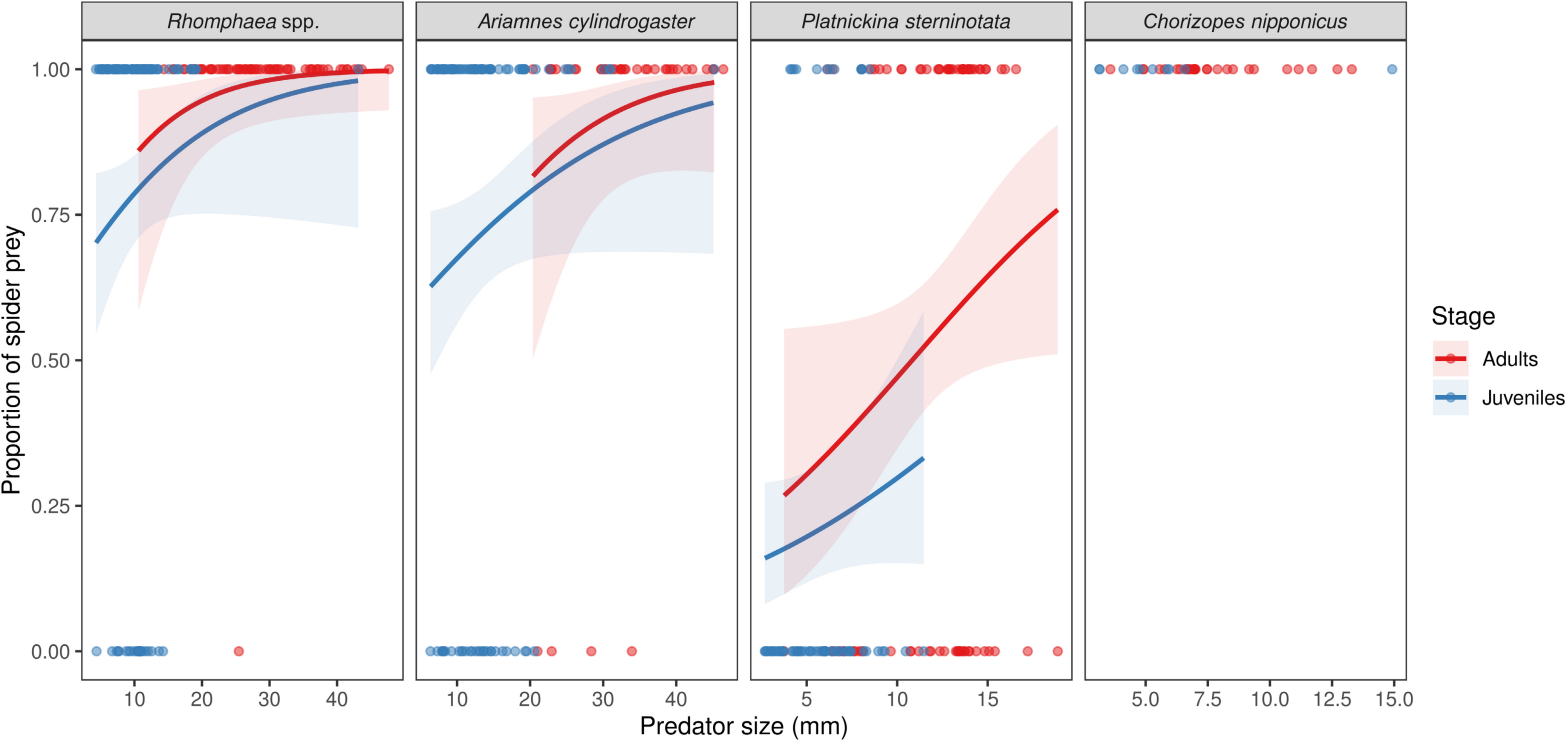
Relationships between predator size (total length in mm) and proportion of spider prey (binary data; non‐dangerous prey = 0, dangerous prey = 1) in araneophagic spiders, considering their life stages (i.e., adults or juveniles). Each point in the plot represents one prey individual, either a spider or not. Lines and shaded areas indicate predicted values and 95% CIs from the GLMs. Although *Chorizopes nipponicus* was not statistically analyzed due to its monophagous diet, points of the prey individuals were shown for comparison.

Predator size, which we hypothesized to be a critical factor for the proportion of spider prey, was included in all of the top models. Species identity, life stage, and quadratic interactions were also included in the top models (Table 1a). The proportion of spider prey is related to predator size. However, the strength and signs of this relationship depend on the species of the predator and their life stage, in addition to the species‐specific manner in their diet and life stage. Specifically, predator size had positive effects on the proportion of spider prey in *Rhomphaea s*pp., *A. cylindrogaster*, and *P. sterninotata* (Figure 3). We also found that *Rhomphaea* and *Ariamnes* kept their proportion of spider prey higher than 0.5; however, it was lower than 0.5 in *Platnickina* when predator size was small (Figure 3). In terms of life stage, the proportion of spider prey in juveniles tended to be lower than that of adults, especially in the case of *P. sterninotata* among the three theridiids (Figure 3). It was shown by comparing the sum of AICc weights that predator size and species were more important factors than life stage, even after considering the interactive effects among the factors (Table 1a: The sum of weights).

**FIGURE 4:**
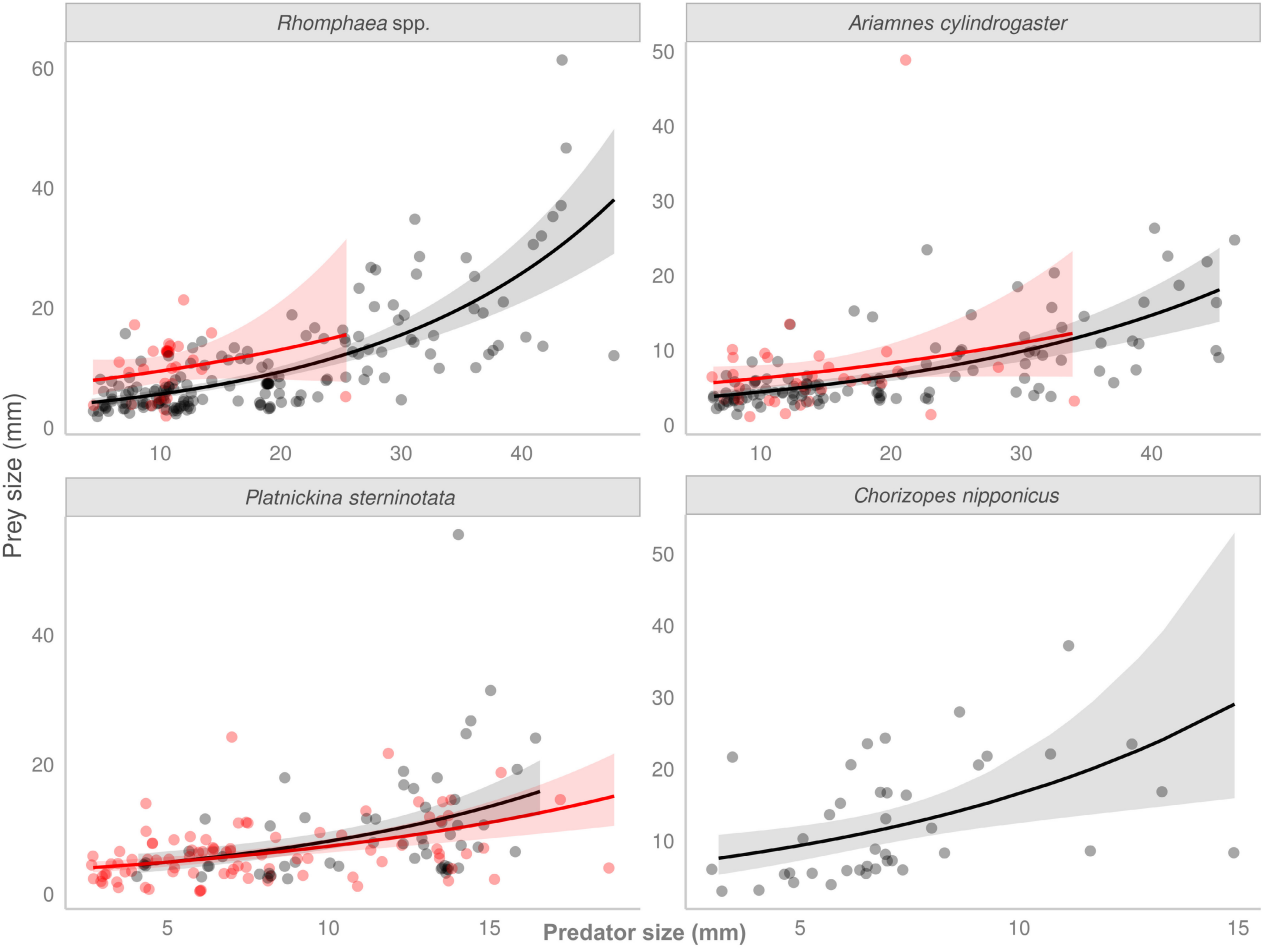
Relationships between prey and predator body size (total length in mm) in four araneophagic spiders. Estimated GLMs with 95% CI are shown. Black circles and red circles indicate spider preys and insect preys, respectively.

For the prey size of the three araneophagic spiders, predator size, predator species, and prey types were equally important because they were included in the best model based on the AICc value, and the sum of the weights of the factors was similarly high (Table 1b). Interactions among these factors were also included in the high‐ranking models, suggesting that the relationships between prey size and predator size depended on species and prey types. Specifically, the prey size of the three spiders that eat both spiders and insects increased with predator size (Figure 4). This was also observed in *C. nipponicus*, which was separately analyzed because of its mono‐prey type. In *Rhomphaea* spp. and *A. cylindrogaster*, prey–predator size relationships differed depending on whether the prey was spiders or not (Figure 4). Specifically, the size of the insect prey of *Rhomphaea* spp. and *A. cylindrogaster* tended to be larger than that of spiders, especially when the predator was relatively small (Figure 4). In *P. sterninotata*, however, prey sizes were similar between spider and insect preys (Figure 4).

## 4 DISCUSSION

In this study, we discovered a novel pattern of ODSs in predator‐eating predators: the diet of araneophagic spiders shifted from mixed (eating both insects and spiders) to a spider‐dominant diet (Figure 3, Figures S2 and S3). The ODS patterns of araneophagic spiders can be distinguished from other reported ODS patterns of predators. For example, a common predator that mostly eats a wide range of prey at lower trophic levels tends to increase its diet breadth as it grows (Mezőfi et al., 2020; Murakami, 1983; Sanders et al., 2015). As the other example, in the case of predators with a narrow diet breadth, they clearly switch between distinct prey taxa, shifting from a specialized to a generalized diet or maintaining a specialized diet by switching between closely related prey taxa (Gajski et al., 2020; Hadson, 1987; Ohba, 2009; Yeargan & Quate, 1996).

The uniqueness of the ODS patterns of spiders in this study may support our hypothesis that predator‐eating predators may shift their focal prey from non‐dangerous to dangerous prey when the predator is under the capture risk. However, there were also interspecific differences in the relationship between prey–predator size and ODS patterns of araneophagic spiders, and effects of life stage per se on the diet composition were detected. Hereafter, we explain how the capture risk of dangerous prey may explain the unique ODS patterns in araneophagic spiders (4.1), then we discuss other potential factors that may affect ODS patterns (4.2).

### 4.1 Size constraints and capture risk

Generally, predators face size constraints and are forced to eat small‐sized prey when they are small juveniles (Scharf et al., 2000). Our data revealed that the four araneophagic spiders showed positive relationships between predator and prey sizes (Figure 4; Table 1b), suggesting that they are under size constraints (Brose, 2010; Brose et al., 2006). In this study, we further expected that small individuals of predator‐eating predators would tend to eat non‐dangerous prey, and the body size of dangerous prey consumed by these predators would be smaller than that of non‐dangerous prey because the capture risk makes it difficult for predator juveniles to subdue large dangerous prey. This hypothesis was supported by the result of gamma GLMs (Figure 4, Table 1b), with which the prey–predator size relationships were detected to have differed significantly between dangerous (i.e., spiders) and non‐dangerous prey (insects). Especially in *Rhomphaea* spp. and *A. cylindrogaster*, the body sizes of spiders predicted by GLMs tended to be smaller than those of insects when the spiders were small (Figure 4; Table 1b). Therefore, such a difference in body size between dangerous and non‐dangerous prey in small predators may indicate that the effect of capture risk on prey selection may be significant for predators. Supporting this, previous studies about the common types of spiders, which mostly eat lower trophic levels but occasionally eat other spiders, also reported that the capture success of dangerous prey (spiders) more rapidly decreases than that of non‐dangerous prey (insects) with an increase in prey–predator size ratio (Michalko & Dvoryankina, 2019; Michalko & Pekár, 2015; Rypstra & Samu, 2005). This implies that those predators are hard to subdue large dangerous prey compared to non‐dangerous ones. Also, an increase in the proportion of spider prey with ontogeny is reported in some non‐araneophagic spiders (Balfour et al., 2003; Bartos, 2011), suggesting that the difficulty in capturing dangerous prey decreases with the growth of the predator. Therefore, our results focusing on the system of predator‐eating predators add strong evidence that the effect of capture risk on prey selection may be one of the critical factors to determine predator's ODS pattern.

### 4.2 Other factors that potentially influence ODS

Among the four araneophagic spiders examined in this study, *C. nipponicus* was the only stenophagous specialist that preyed exclusively on spiders and did not shift prey types during its lifespan (Figure 2). This is surprising because almost all known araneophagic spiders accept both spiders and insects as prey, except for *Nops* species (Caponiidae; García et al., 2018; Hallas, 1988; Jackson & Blest, 1982; Michálek, Lubin, & Pekár, 2019). Such interspecific differences in the ODS patterns among araneophagic spiders may infer that the effect of capture risk on prey utilization is not uniform among predator‐eating predators, and the types of prey‐capturing traits may be linked to such a variation. Concerning prey‐capturing tools, *C. nipponicus* is known as a venom user, while the other spiders we targeted were silk users (Figure 1). In specialized dietary predators, which rely on venom for prey capture, the effect of venom is supposed to be fine‐tuned (Michálek, Kuhn‐Nentwig, & Pekár, 2019; Pekár et al., 2012, 2018). In contrast, such a fine‐tuned venom potentially reduces the capture efficiency of alternative prey (Michálek et al., 2018). Our results suggest that the specialized prey‐capturing tool of *C. nipponicus* may mitigate the capture risk, which enables it to capture its optimal prey (i.e., spider) throughout its ontogeny.

In addition to the capture risks and interspecific differences in capturing tools, we should carefully consider the effects of resource requirements on ODS patterns. As shown in Figure 3 and Table 1, the life stage of the predators somewhat affected the proportion of spider prey, and the tendency was especially clear in *P. sterninotata*. It was suggested by these results that some stage‐dependent factors may also underlie ODS in araneophagic spiders, although the relative importance of the life stage was lower than that of the other main factors (Table 1a). In general, organisms at lower trophic levels (e.g., herbivorous insects) are rich in lipid content, while those at higher trophic levels (e.g., spiders) contain more protein than lipids (Reeves et al., 2021; Wilder et al., 2013). Thus, predators at the top trophic levels tend to suffer from lipid limitation, an essential macronutrient for improving juvenile growth (Jensen et al., 2010; Wiggins & Wilder, 2018). Likewise, our study species may also face such a lipid limitation, and thus juveniles of the three araneophagic spiders may handle the problem by consuming lipid‐rich insects in addition to spider prey, as well as known in mixed diets in generalist predators (Kohl et al., 2015; Lefcheck et al., 2013; Michalko et al., 2020; Oelbermann & Scheu, 2002; Wilder et al., 2013). However, it should be noted that the degree of nutrient limitation should vary among predator taxa, considering that some known predator species, including *C. nipponicus*, can eat specific predator taxa throughout their lifetimes. For instance, juveniles of araneophagic *Portia* spiders possess strong preferences for protein‐rich prey and show lower growth rates under a mixed diet, including both insect and spider, than a spider‐dominant diet (Li & Jackson, 1997; Toft et al., 2010). *Portia* spiders are less affected by lipid limitation and can grow under a protein‐biased diet. Additionally, a stenophagous ant‐eating spider is known to adjust the intake of protein and lipids by selecting a feeding part of the body of the prey (Pekár et al., 2010). Therefore, predator‐eating predators can handle lipid limitations by physiological or behavioral adaptations, and the degree of such adaptations would cause a variety of effects in the life stage itself on ODS, such as our study species showing different ODS patterns. Since we did not assess whether our study species were indeed limited by specific nutrients, further studies are needed to examine the above hypothesis strictly.

## 5 CONCLUSION

We examined ODS patterns in predator‐eating predators and demonstrated that these predators showed a unique ODS pattern, shifting from a generalized (eating both non‐dangerous and dangerous prey) to a specialized diet (a dangerous prey‐dominant diet). This dietary shift is likely due to size constraints and the risks of capturing dangerous prey. Interspecific variations in ODS patterns and prey–predator size relationships suggest that other potential factors, such as prey‐capturing traits and stage‐dependent nutritional requirements, may also be linked to ODS. Overall, the importance of considering both size constraints and capture risk to understand ODS patterns and mechanisms was better emphasized in our study.

## AUTHOR CONTRIBUTIONS

**Yuya Suzuki:** Conceptualization (equal); data curation (equal); formal analysis (equal); investigation (lead); methodology (lead); visualization (lead); writing – original draft (equal). **Mito Ikemoto:** Conceptualization (equal); Data curation (supporting); formal analysis (equal); methodology (supporting); visualization (equal); writing – original draft (supporting); writing – review and editing (lead). **Tomoyuki Yokoi:** Conceptualization (equal); data curation (supporting); formal analysis (supporting); investigation (supporting); methodology (supporting); project administration (lead); supervision (lead); visualization (supporting); writing – original draft (equal); writing – review and editing (lead).

## FUNDING INFORMATION

This research received no specific grant from any funding agency in the public, commercial, or not‐for‐profit sectors.

## CONFLICT OF INTEREST

None of the authors have any conflict of interest.

## Supporting information


Appendix S1

## Data Availability

Data will be available at Dryad Digital Repository.
