# Supplementary material for: The ontogenetic dietary shift from non‐dangerous to dangerous prey in predator‐eating predators under capture risk
Source: Ecol Evol. 2022 Dec 8;12(12):e9609. doi: 10.1002/ece3.9609 (PMC9731918; doi:10.1002/ece3.9609)
Supplement: Supplementary file 1 — Appendix S1 [file ECE3-12-e9609-s001.docx]

**Supplemental Materials**

**
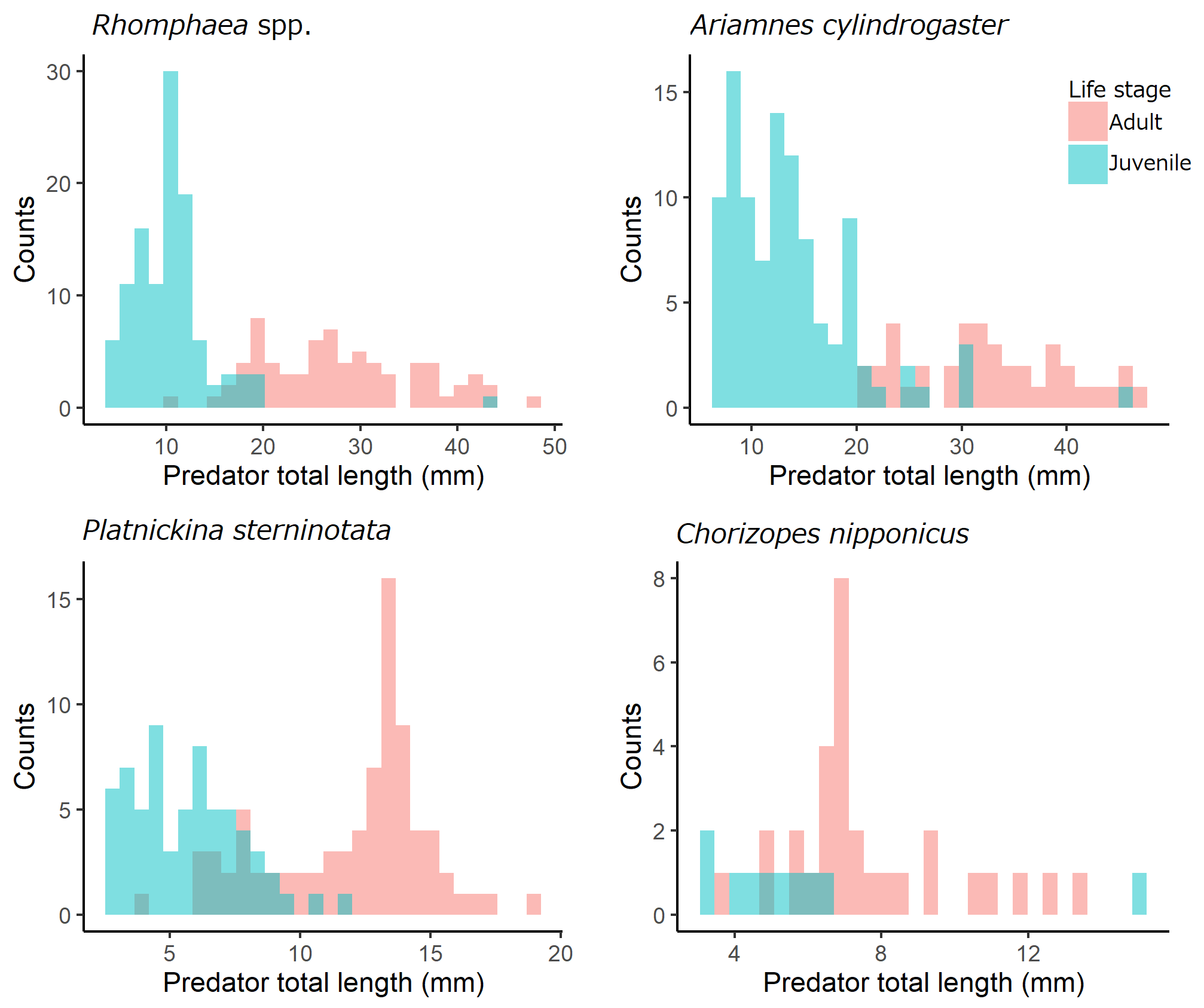
**

**Figure S1.** Distribution of body size (total length) in four araneophagic spiders. Green and pink bars indicate juveniles and adults, respectively.

**
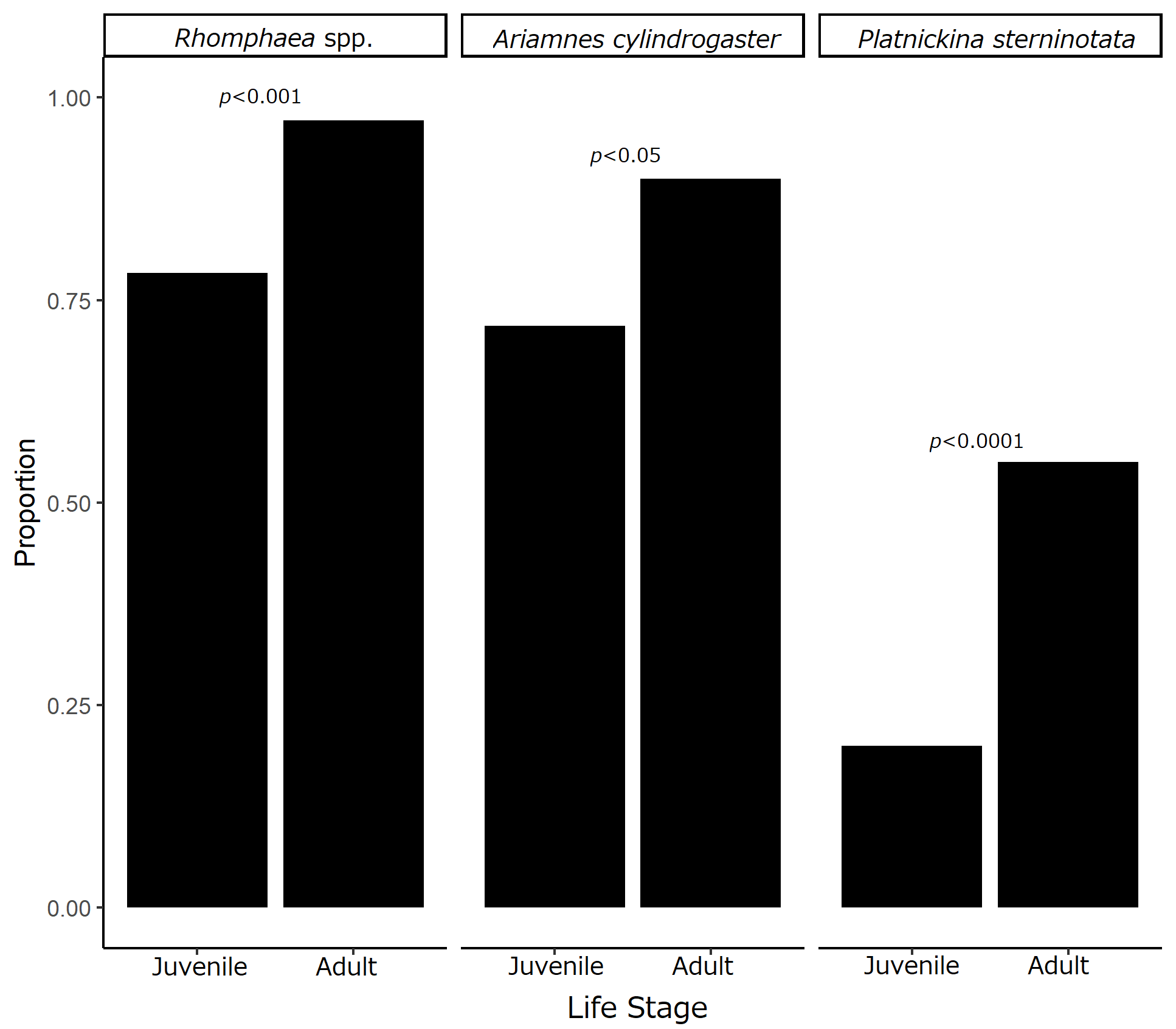
**

**Figure S2.** Proportion of spider prey in the diet of juvenile and adult of the three araneophagic species.

p-values in the plot are calculated by Fisher’s exact probability test (testing differences in proportion of spider prey between juvenile and adult).

**
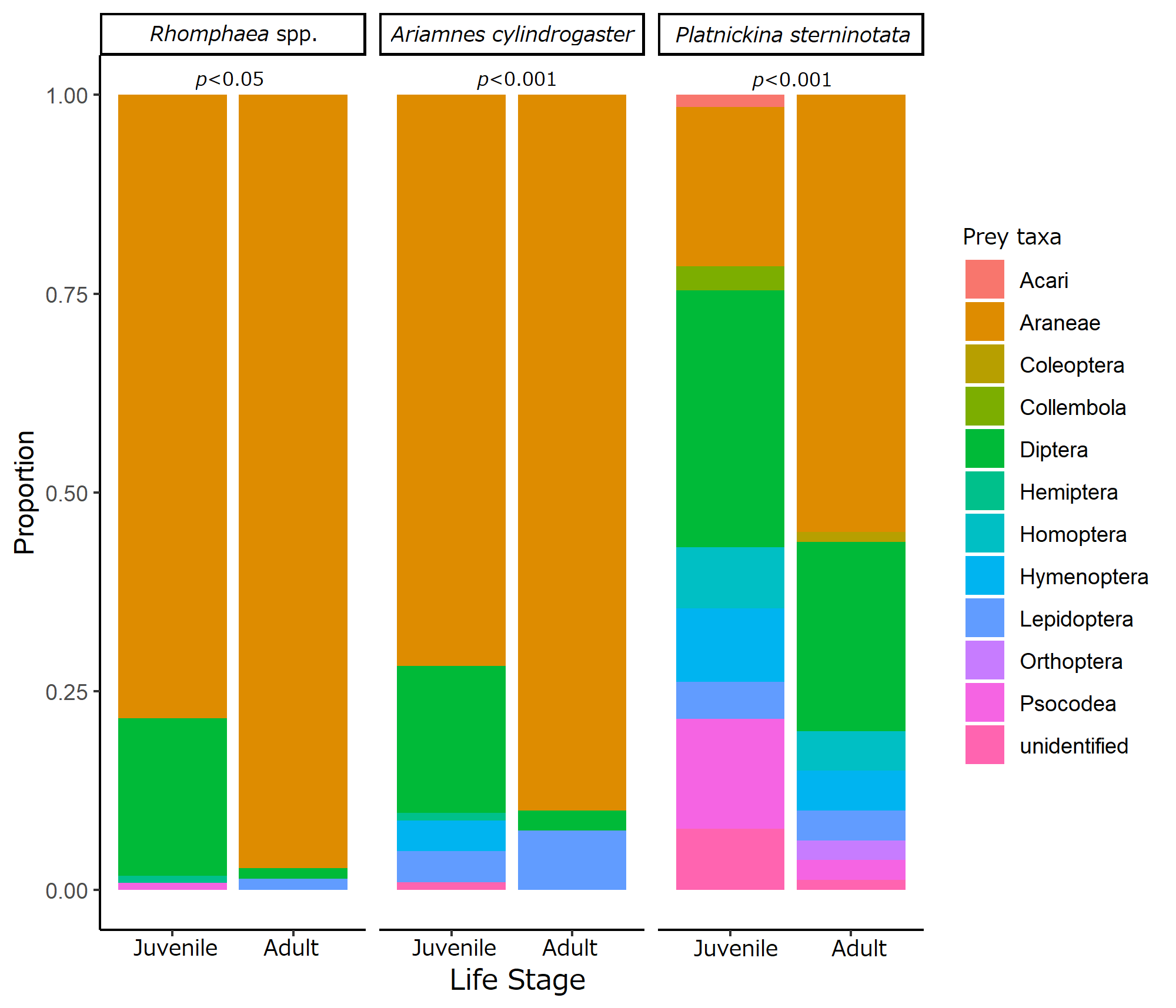
**

**Figure S3.** Proportion of prey taxa (order level) in juvenile and adult of three araneophagic species.

p-values in the plot are calculated by Fisher’s exact probability test (testing differences in prey composition at order level between juvenile and adult).

**
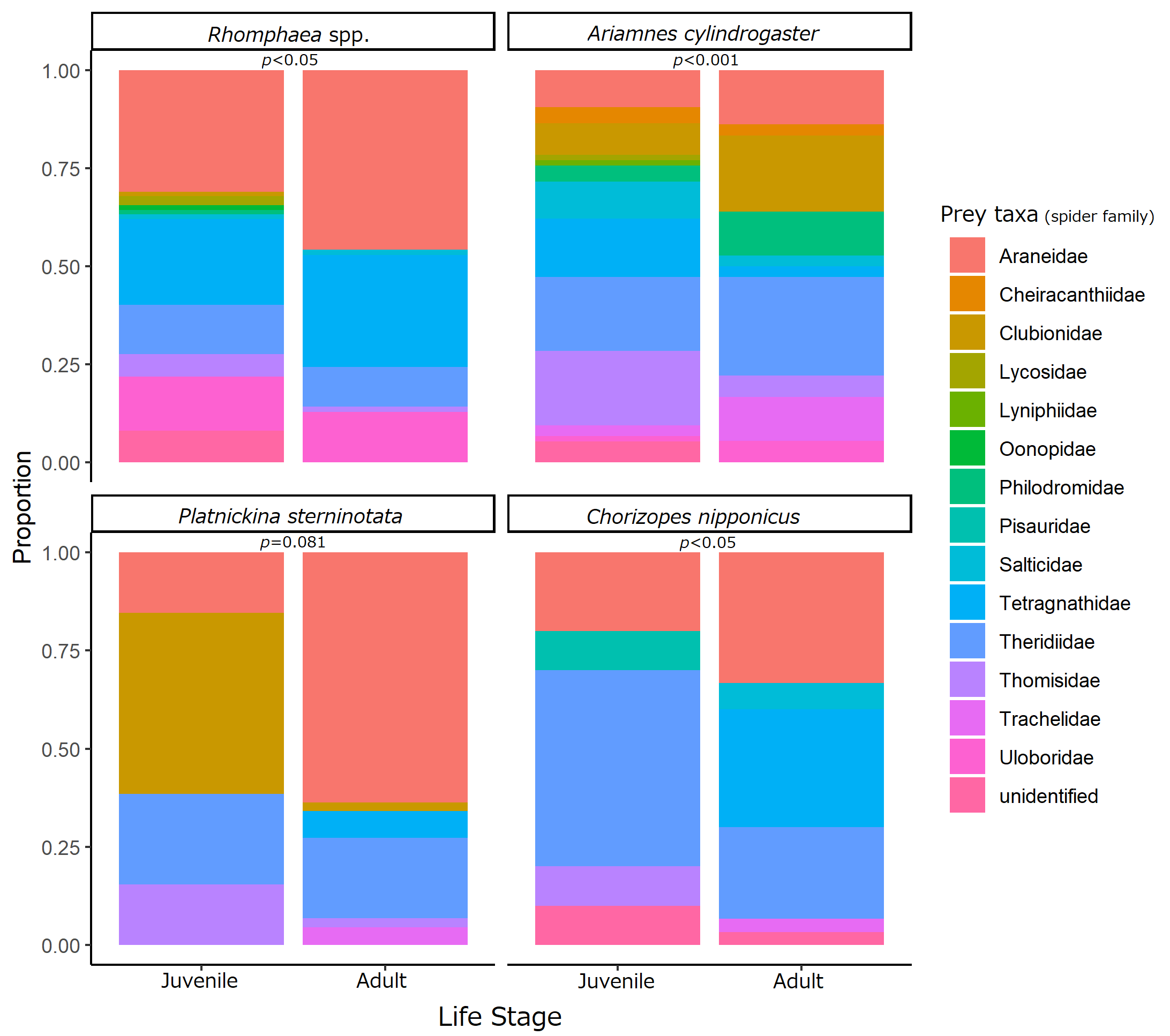
**

**Figure S4.** Proportion of spider prey taxa (family level) in four araneophagic spiders. p-values in the plot are calculated by Fisher’s exact probability test (testing differences in spider prey composition at family level between juvenile and adult).


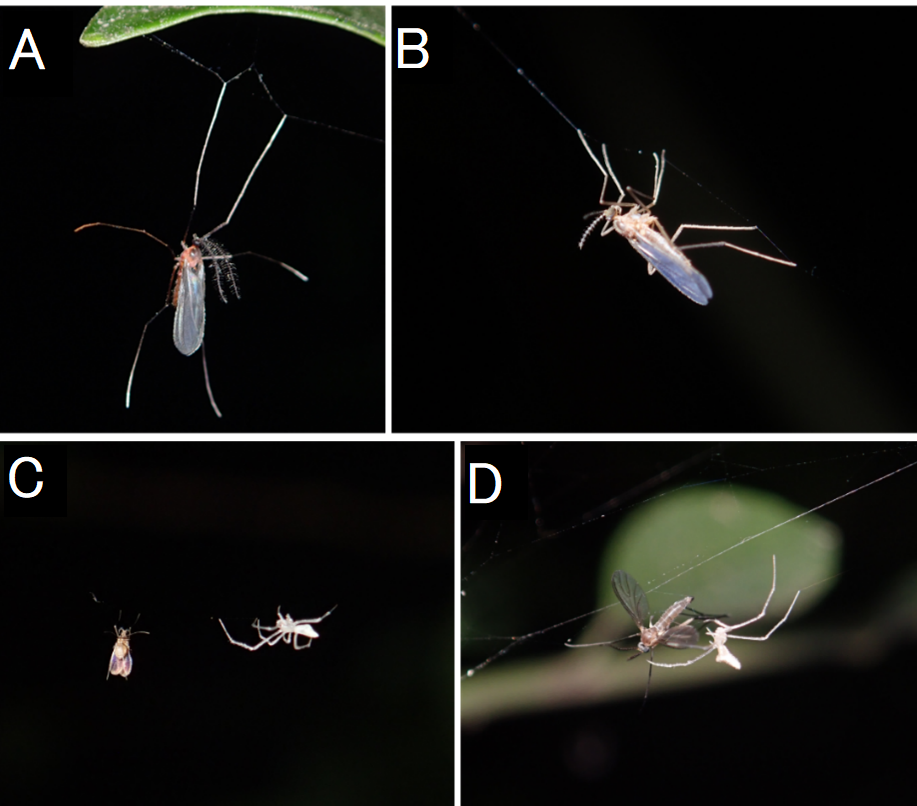
**Figure S5.** Gall midges **(**Diptera: Cecidomyiidae) hanging on line threads (A, B); A juvenile of *Rhomphaea* sp. approaching a gall midge on line threads (C); A juvenile of *Rhomphaea* sp. caught a fungus gnat on line threads (D).

**Table S1**. Prey composition of four araneophagic spiders.

| Prey taxa | | Predator species | | | |
| --- | --- | --- | --- | --- | --- |
|  |  | *Rhomphaea spp.* | *A. cylindrogaster* | *P. sterninotata* | *C. nipponicus* |
| Spider prey | **Araneidae** | 32% | 8% | 21% | 30% |
|  | **Clubionidae** | 1% | 9% | 5% | 0% |
|  | **Eutichuridae** | 0% | 3% | 0% | 0% |
|  | **Linyphiidae** | 0% | 1% | 0% | 0% |
|  | **Oonopidae** | 1% | 0% | 0% | 0% |
|  | **Philodromidae** | 1% | 5% | 0% | 0% |
|  | **Pisauridae** | 0% | 0% | 0% | 3% |
|  | **Salticidae** | 1% | 6% | 0% | 5% |
|  | **Tetragnathidae** | 21% | 8% | 2% | 23% |
|  | **Theridiidae** | 10% | 16% | 8% | 30% |
|  | **Thomisidae** | 3% | 11% | 2% | 3% |
|  | **Trachelidae** | 0% | 4% | 1% | 3% |
|  | **Uloboridae** | 11% | 2% | 0% | 0% |
|  | **unidentified spiders** | 4% | 3% | 0% | 5% |
|  | **Total spiders** | 86% | 77% | 39% | 100% |
| Non-spider prey | **Acari** | 0% | 0% | <1% | 0% |
|  | **Coleoptera (larvae)** | 0% | 0% | <2% | 0% |
|  | **Collembola** | 0% | 0% | <3% | 0% |
|  | **Diptera** | 13% | 14% | 28% | 0% |
|  | **Homoptera** | <1% | <1% | 6% | 0% |
|  | **Hymenoptera** | 0% | 3% | 7% | 0% |
|  | **Lepidoptera (larvae)** | <1% | 5% | 4% | 0% |
|  | **Orthoptera (nymphs)** | 0% | 0% | <1% | 0% |
|  | **Psocoptera** | <1% | 0% | 8% | 0% |
|  | **Unidentified arthropods** | 0% | <1% | 4% | 0% |
|  | **Total number of observations** | 183 | 143 | 145 | 40 |

**Table S2**. Diet breadth (Shannon’s H’) for prey order and spider prey family in juvenile and adult of the four araneophagic spiders. Diet breadth for prey order is not shown in *C. nipponicus* because it only preyed on spiders.

| Species | Diet width for prey order (H’) | | Diet width for spider prey family (H’) | |
| --- | --- | --- | --- | --- |
|  | juvenile | adult | juvenile | adult |
| *Rhomphaea* spp. | 0.60 | 0.15 | 1.89 | 1.33 |
| *Ariamnes cylindrogaster* | 0.90 | 0.39 | 2.25 | 2.05 |
| *Platnickina sterninotata* | 1.89 | 1.39 | 1.27 | 1.11 |
| *Chorizopes nipponicus* | — | — | 1.36 | 1.47 |
